# DTCMMA: Efficient Wind-Power Forecasting Based on Dimensional Transformation Combined with Multidimensional and Multiscale Convolutional Attention Mechanism

**DOI:** 10.3390/s25154530

**Published:** 2025-07-22

**Authors:** Wenhan Song, Enguang Zuo, Junyu Zhu, Chen Chen, Cheng Chen, Ziwei Yan, Xiaoyi Lv

**Affiliations:** 1 School of Computer Science and Technology, Xinjiang University, Urumqi 830046, China; songwenhan101@163.com (W.S.); 107552304224@stu.xju.edu.cn (J.Z.); 2 School of Intelligence Science and Technology, Xinjiang University, Urumqi 830017, China; zeg@xju.edu.cn; 3 Department of Electronic Information, Tsinghua University, Beijing 100084, China; 4 School of Software, Xinjiang University, Urumqi 830091, China; chen_chen@stu.xju.edu.cn (C.C.); chenchengoptics@gmail.com (C.C.); ziwei13579@163.com (Z.Y.)

**Keywords:** wind-power forecasting, dimensional transformation, convolutional attention mechanism, deep learning

## Abstract

With the growing global demand for clean energy, the accuracy of wind-power forecasting plays a vital role in ensuring the stable operation of power systems. However, wind-power generation is significantly influenced by meteorological conditions and is characterized by high uncertainty and multiscale fluctuations. Traditional recurrent neural network (RNN) and long short-term memory (LSTM) models, although capable of handling sequential data, struggle with modeling long-term temporal dependencies due to the vanishing gradient problem; thus, they are now rarely used. Recently, Transformer models have made notable progress in sequence modeling compared to RNNs and LSTM models. Nevertheless, when dealing with long wind-power sequences, their quadratic computational complexity (O(L^2^)) leads to low efficiency, and their global attention mechanism often fails to capture local periodic features accurately, tending to overemphasize redundant information while overlooking key temporal patterns. To address these challenges, this paper proposes a wind-power forecasting method based on dimension-transformed collaborative multidimensional multiscale attention (DTCMMA). This method first employs fast Fourier transform (FFT) to automatically identify the main periodic components in wind-power data, reconstructing the one-dimensional time series as a two-dimensional spatiotemporal representation, thereby explicitly encoding periodic features. Based on this, a collaborative multidimensional multiscale attention (CMMA) mechanism is designed, which hierarchically integrates channel, spatial, and pixel attention to adaptively capture complex spatiotemporal dependencies. Considering the geometric characteristics of the reconstructed data, asymmetric convolution kernels are adopted to enhance feature extraction efficiency. Experiments on multiple wind-farm datasets and energy-related datasets demonstrate that DTCMMA outperforms mainstream methods such as Transformer, iTransformer, and TimeMixer in long-sequence forecasting tasks, achieving improvements in MSE performance by 34.22%, 2.57%, and 0.51%, respectively. The model’s training speed also surpasses that of the fastest baseline by 300%, significantly improving both prediction accuracy and computational efficiency. This provides an efficient and accurate solution for wind-power forecasting and contributes to the further development and application of wind energy in the global energy mix.

## 1. Introduction

**Background:** With the continuous growth of global energy demand and the increasing emphasis on environmental protection, the development and utilization of clean energy have become central to the current global energy transition. As a renewable and pollution-free clean energy source, wind-power generation plays a vital role in the transformation of the global energy structure. According to the International Energy Agency (IEA) [1], global installed wind-power capacity has seen rapid growth in recent years, making wind power the third-largest source of electricity worldwide. However, wind-power generation is significantly affected by natural environmental factors and is characterized by high uncertainty and volatility. Random changes in meteorological conditions such as wind speed, wind direction, temperature, and other factors, as well as geographic environment, cause wind-power power output to show complex spatial and temporal variation characteristics, which brings significant challenges to the stable operation of power systems and energy management. Accurate wind-power forecasting is thus of great importance for the reliable operation of power systems, the optimization of energy dispatch, and the sustainable development of the wind-power industry. From the perspective of grid dispatching, precise forecasting results can assist operators in formulating reasonable generation plans in advance, optimizing the operational state of the power system, enhancing the grid’s capability to integrate wind energy, and reducing the demand for backup power caused by wind-power fluctuations, thereby lowering overall operational costs. For the operation of wind farms, accurate power prediction can help optimize the maintenance and management strategy of wind turbines, arrange equipment maintenance in advance, reduce the failure rate, and improve the power-generation efficiency and economic benefits. Furthermore, in the context of electricity markets, accurate forecasting data provides strong support for wind farms to participate in power trading, enhancing their competitiveness in the market. Therefore, improving the accuracy and reliability of wind-power forecasting has become a key step toward promoting the further development of the wind-power industry.

**Challenge**: In the field of wind-power forecasting, researchers primarily adopt physical models and statistical models for prediction. Physical models rely on numerical weather prediction and various meteorological parameters to directly characterize the impact of atmospheric changes on wind-power output by accurately simulating atmospheric physical processes. Statistical models, on the other hand, focus on time-series analysis using historical wind-power data to uncover trends and patterns in power fluctuations, thereby enabling forecasting [2]. However, existing physical models have certain limitations in capturing the fine-grained fluctuation characteristics of wind power [3]. They typically require large volumes of meteorological data and often lack adaptability to complex and dynamic real-world weather conditions. While statistical models can effectively fit trends in historical data, wind-power data often consists of multiple feature dimensions, such as wind speed at different heights, wind direction from various angles, temperature, humidity, and other meteorological features, as well as operational features of wind turbines such as rotor speed and power output. Current models based on recurrent neural networks (RNN) and their variants, as well as Transformer and its improved versions, are predominantly one-dimensional. When dealing with these multidimensional features, they often concatenate feature dimensions directly or apply simple linear transformations for fusion, lacking in-depth modeling of the correlations among different features. As a result, they fail to fully exploit the hidden multiscale periodic patterns and local spatial correlations within the data.

**Figure 1:**
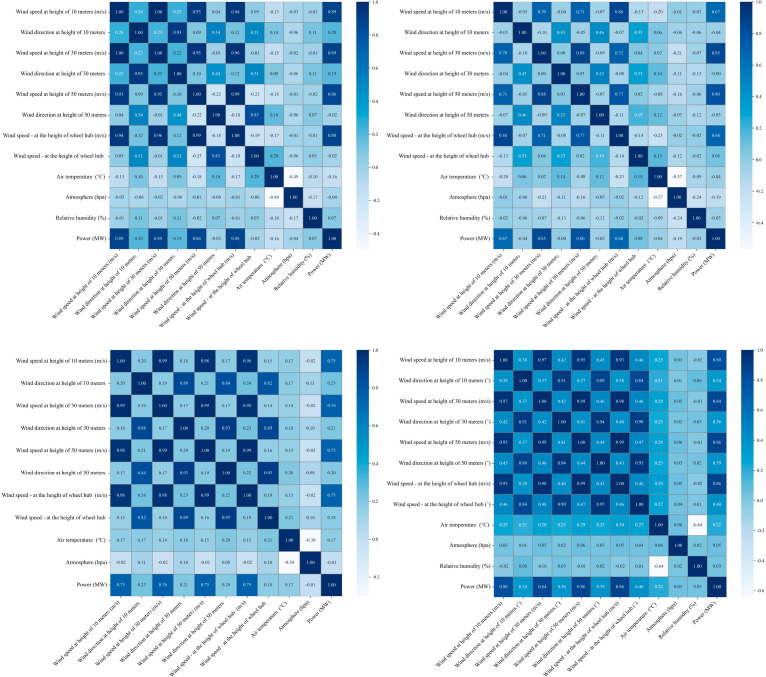
Visualization of inter-channel correlations in the wind-power dataset from a wind farm in Xinjiang, China.

**Observations and Reflections:** To better investigate the correlations among different features, we conducted a visualization experiment on inter-channel correlations. Specifically, we calculated the Pearson correlation coefficient matrix among various feature channels in the wind-power dataset and presented it in the form of a thermodynamic diagram, as shown in Figure 1. The experiment revealed that significant correlations exist between many features, while others exhibit weaker relationships. However, existing models fail to fully exploit these correlations for effective feature fusion and learning. For instance, wind speed and turbine power output demonstrate a strong positive correlation, but models may not adequately capture this association during training, resulting in suboptimal prediction accuracy for wind-power fluctuations. By visualizing and analyzing the correlation between different feature channels of wind-power data, it was found that there is a significant correlation between meteorological parameters such as wind speed, temperature, humidity, and wind-power output, but the existing one-dimensional model cannot make full use of this correlation information. Based on this observation, we adopted the dimension transformation concept from the TimesNet model [4] to reconstruct the one-dimensional time series as a two-dimensional representation. Tailored to the characteristics of wind-power time-series data, we designed a collaborative multidimensional multiscale attention (CMMA) mechanism. This model can better capture the complex dependencies in wind turbine time-series data at the two-dimensional level and effectively identify multiscale feature patterns. Compared to the Transformer family and TimesNet, our model demonstrates significantly higher time efficiency and stronger application potential.

**RNN-based prediction model:** RNN and its variants, such as LSTM [5] and GRU [6], offer certain advantages in handling sequential data, as they are capable of capturing short-term dependencies in time series. LSTM alleviates the vanishing gradient problem of traditional RNNs [7] by introducing gating control mechanisms, enabling the model to learn longer temporal dependencies. GRU [8], built upon LSTM, further simplifies the architecture by reducing the number of parameters and improving training efficiency [9]. However, these models still face several limitations when processing long sequences [6]. Firstly, RNN-based models are prone to gradient vanishing or explosion during training, resulting in slow convergence and unstable learning [10]. Secondly, for wind-power data with complex periodicity and multiscale characteristics, RNNs struggle to simultaneously capture short-term fluctuations and long-term trends [11,12]. Thirdly, when dealing with datasets containing a large number of variable features, RNN models often fail to effectively learn the correlations among different features. They tend to process each feature independently, overlooking the interactions between features, which ultimately limits the improvement of forecasting accuracy.

**Transformer-based prediction model**: Since its introduction, the Transformer model has achieved remarkable success across a wide range of sequence modeling tasks [13] thanks to its self-attention mechanism, which enables the parallel computation of inter-feature relationships and excels at capturing global dependencies in sequences. In the field of wind-power forecasting, several Transformer-based models—such as Informer [14] and Autoformer [15]—have emerged. Informer introduces a ProbSparse attention mechanism and a generative sequence distillation technique to reduce computational complexity while enhancing long-sequence modeling capabilities. Autoformer combines an autocorrelation mechanism with time-series decomposition methods. As one of the latest representatives, iTransformer [16] innovatively proposes a “channel-first” inverted architecture to optimize multivariate time-series modeling, improving the recognition of complex periodic patterns. Nevertheless, there are still some urgent problems with the Transformer series of models. On the one hand, the traditional Transformer’s self-attention mechanism has O(L^2^) time complexity, which consumes huge computational resources when dealing with ultra-long sequences, limiting its application on large-scale wind-power datasets; on the other hand, its ability to capture local features in the sequence is relatively weak, and it is easy to submerge the local strongly dependent signals in the global noise, which leads to insufficient expression of the detailed features of wind-power data. In addition, when dealing with multivariate feature datasets, most of the existing Transformer series models are one-dimensional models, which are directly spliced with each feature dimension or fused by simple linear transformations, lacking in the deep mining and modeling of the correlation between different features and failing to fully excavate the implied multiscale periodic patterns and local spatial correlations in the data.

**Prediction model based on dimension transformation and hybrid strategy**: In recent years, several innovative modeling paradigms have emerged in the field of time-series forecasting [17], among which TimesNet [4] and TimeMixer [18] stand out as representative approaches, offering new perspectives on feature representation and dependency modeling for temporal data. TimesNet, based on the inherent multi-periodic nature of time series, was the first to propose a modeling paradigm that transforms one-dimensional (1D) time series into two-dimensional (2D) representations [14]. The method identifies the major periodic components of the time-series data by fast Fourier transform and reconstructs the 1D sequence into a 2D tensor according to different cycle lengths, thus modeling complex time-varying patterns within and between cycles simultaneously in 2D space. TimesNet adopts the standard Inception block [19] as a feature extractor, which is capable of capturing multiscale spatiotemporal dependencies. The advantage of this dimension transformation strategy is the ability to explicitly encode the implicit periodic structure of the time-series data in the spatial dimension, which provides a richer representation space for the learning of complex temporal patterns. TimeMixer, on the other hand, employs a decomposition-mixing forecasting architecture, which decomposes the time-series forecasting task into two complementary modeling phases, namely, the mixing of past information and the mixing of future information. The method is able to capture both short-term fluctuations and long-term trends through a multiscale patch mixing mechanism that effectively integrates historical information at different time scales.

**Limitations and challenges of existing methodologies**: Despite the progress made by the above methods within their respective design concepts, there are still significant limitations and challenges in the specific domain of wind-power prediction. First is the limitation of dimensional modeling. Mainstream models such as RNNs, LSTM models, and Transformers are fundamentally based on one-dimensional (1D) sequence modeling paradigms. When dealing with the multidimensional meteorological features present in wind-power data—such as wind speed, wind direction, temperature, and humidity—these models typically adopt simple feature concatenation or linear transformations for fusion. This approach fails to sufficiently capture the complex interdependencies and interaction patterns among different feature dimensions. Although iTransformer introduces the concept of inter-variable attention, its inverted architecture still struggles to balance computational efficiency and feature expressiveness when modeling long sequences. Second is the issue of geometric adaptability. While TimesNet pioneered a 2D modeling paradigm, it employs standard Inception blocks with square convolutional kernels (e.g., 3 × 3, 5 × 5). This design leads to severe spatial padding issues when handling the elongated geometric shapes of the 2D data reconstructed from sequences with high length-to-period ratios. In such cases, the reshaped data forms narrow and long stripe-like structures, requiring excessive padding with traditional square kernels. This not only increases the computational burden but may also introduce noise, hindering the effective learning of critical temporal patterns. TimeMixer, although adopting a multiscale mixing strategy, primarily focuses on temporal multiscale modeling. It lacks the capability for coordinated modeling of spatial correlations, inter-channel dependencies, and pixel-level detail features, all of which are prominent in wind-power data. Existing methods lack a unified multidimensional attention framework for collaborative feature selection and enhancement at different abstraction levels, such as channel, space, and pixel [20,21]. Most critically, it is difficult to balance computational efficiency and accuracy. The O(L^2^) complexity of the Transformer family of models is inefficient when dealing with long sequences of wind-power data, while existing sparse attention mechanisms reduce computational complexity, albeit often at the expense of the ability to capture local temporal patterns. How to achieve significant improvement in computational efficiency while maintaining high prediction accuracy remains a core challenge in the field of wind-power prediction.

**Main work of this paper**: Aiming at the problems of complex spatiotemporal dependencies, nonlinear dynamics, and long- and short-term dependencies in WPF, we propose an efficient wind-power prediction method based on dimension transformation combined with a multidimensional and multiscale attention mechanism. We follow the concept of dimensional transformation introduced in a recent paper [4], in which dimensional transformation is performed on the input data to better mine the texture information of the data, and we design a multidimensional attention mechanism for the multidimensional features of wind-power data, including three attention modules, namely, pixel, channel, and space. The pixel-attention module generates the attention map through a two-layer convolution operation, highlights important pixel regions, suppresses unimportant pixel points, and accurately captures local detail features. The channel attention module uses adaptive pooling and 1 × 1 convolution to learn inter-channel correlation, generate channel weights, strengthen key channel features, and suppress redundant information. The spatial-attention module adopts a multi-branch structure to capture multiscale spatial information using depth-separable convolutions of different sizes, extracts short-term and long-term temporal patterns, and fuses the features through a 1 × 1 convolution to generate the final spatial-attention map. Specifically, channel attention answers the question of “what features are important”, spatial attention solves the question of “where information is important”, and pixel attention precisely controls the question of “how important”. This 3D synergistic design allows the model to perform feature selection and enhancement at different levels of abstraction, which significantly improves the quality of the feature representation. The final feature fusion module deeply fuses the outputs of these three modules, integrating feature information at different levels to produce a comprehensive feature representation. This allows our model and its efficient approach to fully exploit the relevance of wind-power data across different dimensions and scales, enhancing key features and suppressing redundant information. The main contributions are summarized as follows:

- Our research proposes the DTCMMA model framework, specifically designed to address the unique challenges of wind-power forecasting and related energy indicator prediction tasks. The model fully considers the inherent strong periodic fluctuation characteristics and multidimensional coupling attributes of wind-farm time-series data, effectively resolving the modeling limitations of traditional methods when processing complex time-varying patterns through innovative architectural design, providing a systematic technical solution for the wind-power forecasting domain.
- Our research breakthrough transforms the time-series modeling perspective from traditional one-dimensional linear processing to two-dimensional spatial representation, achieving deep mining of implicit feature patterns. We innovatively construct a three-dimensional collaborative attention architecture based on channel importance identification, spatial relationship capture, and pixel-level fine regulation. This mechanism not only demonstrates excellence in wind-power forecasting tasks but also exhibits strong generalization capabilities for cross-domain applications, providing a universal technical framework for multivariate time-series prediction problems.
- Long-sequence forecasting experiments conducted on four wind-farm datasets, four power transformer datasets, and one weather dataset validate the effectiveness of the proposed model. Compared with eight state-of-the-art (SOTA) models, our method achieves relative improvements ranging from 4.18% to 81.02%, while also achieving a 2- to 5-fold increase in time efficiency over baseline models.

## 2. Theory and Methodology

### 2.1. Overall Framework

**Figure 2:**
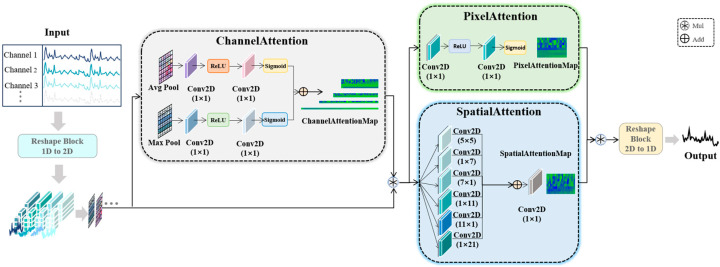
Overall framework diagram of the DTCMMA model.

The wind-power prediction problem aims to predict the power output for the next H time steps based on a historical power sequence X_input ∈ R^(L × D), where L denotes the length of the input sequence and D is the feature dimension. Traditional one-dimensional time-series modeling methods have limitations in capturing the complex time-series dependencies of time-series data such as wind power, which is characterized by strong periodicity and multiscale variations. To address the above challenges, this paper proposes the DTCMMA (dimension transformation with cooperative multidimensional multiscale attention) model. The core idea of the model is to reconstruct the original one-dimensional time series into a two-dimensional representation through the dimension transformation operation and then utilize a cooperative multidimensional multiscale attention mechanism to capture the spatiotemporal dependencies at different granularities. The overall architecture consists of two core components: the dimension transformation module and the collaborative multidimensional multiscale attention mechanism (CMMMA). The details are shown in Figure 2.

### 2.2. Dimension Conversion Module

Learning from the periodicity characteristics of time series, we first identify the major periodic components in the data by fast Fourier transform [22]. Specifically, the Fourier transform is applied to the input sequence X_input as follows:

(1) $$A=Mean(|FFT(X_{input})|)$$

Here, A represents the average amplitude of each frequency component. The Top-K selection operation is applied to obtain the k most significant frequency components $\left\{f_{1},f_{2},\ldots ,f_{k}\right\}$, based on which the corresponding period lengths are determined:

(2) $$p_{i}=\lfloor \frac{L}{f_{i}}\rfloor ,i\in \{1,2,\ldots ,k\}$$

**Figure 3:**
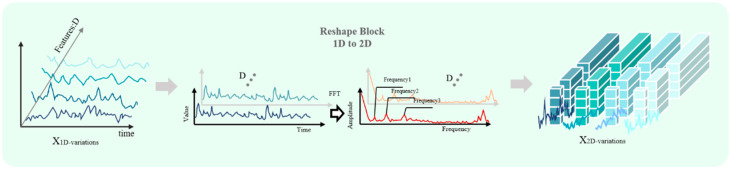
Schematic diagram of the dimension transformation module based on the fast Fourier transform technique. Lines of different colors represent variable sequences from different channels.

Based on the identified cycle information, the one-dimensional time series is reconstructed into a two-dimensional representation. For each period p_i, the following transformation is performed:

(3) $$X_{2D}^{\left(i\right)}={Reshape}_{p_{i},L/p_{i}}(Padding(X_{input}))$$

where the Padding(·) operation ensures that the length of the sequence is divisible by the period p_i, $X_{2D}^{\left(i\right)}\in R^{H\times W\times D}$ is the reconstructed two-dimensional representation, and H = L/p_i_, W = p_i_. This dimensional transformation strategy enables the explicit encoding of intra-periodic and inter-periodic variability patterns of the time series in a two-dimensional spatial structure. We summarize this process in Figure 3.

### 2.3. Collaborative Multidimensional Multiscale Attention Mechanism (CMMMA)

The CMMMA module is designed with three synergistic attention mechanisms that work together to capture the multiscale dependencies of timing data in channel, spatial, and pixel dimensions, respectively.

#### 2.3.1. Adaptive Dual Path Channel Attention

The design goal of the channel attention mechanism is to learn the complex interdependencies among different feature channels and adaptively adjust the contribution weights of each channel to the final prediction task. Considering that different meteorological features (e.g., wind speed, wind direction, temperature, etc.) in wind-power data have varying importance and interaction patterns, we adopt a dual-path aggregation strategy to obtain a more comprehensive and robust channel-level feature representation. Specifically, we simultaneously utilize two different feature aggregation approaches, global mean pooling and global maximum pooling:

(4) $$F_{avg}=GAP(X_{2D})=\frac{1}{H\times W}\sum_{i=1}^{H}\sum_{j=1}^{W}X_{2D}(i,j,:)$$

(5) $$F_{max}=GMP\left(X_{2D}\right)=\underset{i,j}{max}\;X_{2D}\left(i,j,:\right)$$

Global mean pooling captures the overall statistical properties within a channel, reflecting the overall activation level of the features in that channel [23], while global maximum pooling focuses on the salient activation regions within a channel, which helps to identify critical temporal patterns. With dual-path feature extraction, the model is able to consider both the average and peak responses of the features, resulting in richer channel-level semantic information.

Subsequently, the dual-path features are nonlinearly transformed by a shared multilayer perceptron network:

(6) $$A_{avg}=W_{2}(ReLU(W_{1}(F_{avg})))$$

(7) $$A_{max}=W_{2}(ReLU(W_{1}(F_{max})))$$

where $W_{1}\in R^{D/r\times D}$ and $W_{2}\in R^{D\times D/r}$ are the learnable parameters, and r is the channel compression ratio, which is used to control the model complexity. The final channel attention weights are obtained by fusing the dual path information and applying a Sigmoid activation function:

(8) $$M_{c}=\sigma (A_{avg}+A_{max})$$

This design enables the channel attention mechanism to dynamically enhance the meteorological feature channels that are important for the power prediction task while suppressing the influence of noise or redundant features.

#### 2.3.2. Adaptive Multiscale Spatial Attention

The spatial-attention module is specifically designed to capture the importance distribution of different spatial locations in a 2D time-series representation [24], as well as the long- and short-range dependencies between locations. In our replication experiments with the TimesNet model, we found that 2D data reconstructed based on period splitting tends to exhibit irregular geometries, especially when the ratio of sequence length to period length is large, and the reconstructed data often exhibits a significant long bar shape instead of a standard square matrix. This geometric property makes the square convolutional kernels, such as 3 × 3, 5 × 5, etc., used in the traditional Inception block [25], face a serious space-filling problem when performing feature extraction, and a large percentage of the filled area will not only dilute the effective feature information but may also introduce additional noise interference, which affects the model’s effective learning of temporal patterns.

To address the above problems, we propose an adaptive multiscale spatial-attention mechanism, which employs an asymmetric convolutional kernel design to adapt to the special geometrical structure of two-dimensional time-series data. Specifically, we first establish the underlying local spatial receptive field by a 5 × 5 depth-separable convolution [26]:

(9) $$F_{base}={DWConv}_{5\times 5}(X_{2D})$$

Then, based on this base feature map, we design three sets of cross-direction asymmetric convolutions to capture spatial dependencies at different scales:

(10) $$F_{cross1}={DWConv}_{7\times 1}({DWConv}_{1\times 7}(F_{base}))$$

(11) $$F_{cross2}={DWConv}_{11\times 1}({DWConv}_{1\times 11}(F_{base}))$$

(12) $$F_{cross3}={DWConv}_{21\times 1}({DWConv}_{1\times 21}(F_{base}))$$

The advantages of this cross-convolution design are that (1) the asymmetric convolution kernel can better adapt to the geometrical properties of long-strip data, significantly reducing the need for padding operations [27]; (2) the cross-directional convolution is able to capture spatial dependencies in the horizontal and vertical directions, respectively, which corresponds to intra-periodic and inter-periodic patterns of variation in the time-series data; and (3) the multiscale feel design allows the model to simultaneously capture short-, medium-, and long-term spatial and temporal correlations.

The final spatial-attention features are obtained by adaptive fusion of multiscale information:

(13) $$M_{s}={Conv}_{1\times 1}(F_{base}+F_{cross1}+F_{cross2}+F_{cross3})$$

Among them, the 1 × 1 convolution plays the role of a feature dimension adjustment and information fusion mechanism to ensure the effective integration of features at different scales.

#### 2.3.3. Fine-Grained Pixel-Level Attention

The pixel-attention mechanism accurately regulates the importance of features at each spatial location at the fine-grained level, which is particularly critical for wind-power prediction tasks, where power values at different moments may have significantly different predictive values. The mechanism learns the pixel-level distribution of attention weights through a lightweight but efficient convolutional neural network structure.

For the specific implementation, we first reduce the computational complexity through channel compression operations while maintaining feature expressiveness:

(14) $$F_{compressed}=ReLU({Conv}_{1\times 1}^{C\to C/8}(X_{2D}))$$

where the channel compression ratio of *C*/8 significantly reduces the number of parameters while maintaining sufficient expressive power. Subsequently, the features are projected to a single-channel attention map by another 1 × 1 convolution:

(15) $$A_{pixel}={Conv}_{1\times 1}^{C/8\to 1}(F_{compressed})$$

The final normalized pixel-level attention weights are generated by the Sigmoid activation function:

(16) $$M_{p}=\sigma (A_{pixel})$$

The design enables the model to learn the degree of importance of each spatiotemporal location to the prediction task, thus enabling fine-grained feature enhancement [28]. In particular, for critical moments in the wind-power data (e.g., power mutation points, extreme points, etc.), pixel attention can automatically assign higher weights to enhance the contribution of this critical information to the prediction results.

#### 2.3.4. Hierarchical Synergistic Attention Fusion

The three attention mechanisms perform feature enhancement through a well-designed hierarchical and synergistic approach, which follows the principle of feature processing from global to local, and from coarse-grained to fine-grained.

First, channel attention is applied at the global level for importance recalibration of feature channels:

(17) $$X_{ca}=M_{c}⊙X_{2D}$$

The early application of channel attention enables the filtering of unimportant feature channels at the initial stage of feature processing, laying a solid foundation for subsequent spatial and pixel-level operations.

Based on the channel-enhanced features, the outputs of spatial attention and pixel attention are computed, respectively:

(18) $$X_{sa}=\mathrm{SpatialAttention}(X_{ca})$$

(19) $$X_{pa}=\mathrm{PixelAttention}(X_{ca})$$

Eventually, the synergistic fusion of both spatial and pixel-dimensional attention is realized through element-by-element product operations:

(20) $$X_{out}=X_{sa}⊙X_{pa}$$

This hierarchical synergistic fusion design offers the following advantages:

(1) Attention mechanisms at different abstraction levels can capture feature importance at varying granularities, resulting in complementary feature enhancement effects.
(2) The progressively refined processing pipeline ensures the orderly transmission and layer-by-layer enhancement of feature information.
(3) The synergistic fusion mechanism enables the model to comprehensively integrate multidimensional attention information, thereby better addressing the complexity demands of the wind-power forecasting task.

#### 2.3.5. Adaptive Aggregation and Output Mapping

For the feature representation $\left\{X_{out}^{\left(1\right)},X_{out}^{\left(2\right)},\ldots ,X_{out}^{\left(k\right)}\right\}$ obtained from multiple cycle reconstruction, adaptive weighted fusion based on frequency domain importance is used:

(21) $$X_{final}=Reshape(\sum_{i=1}^{k}w_{i}X_{out}^{\left(i\right)}+X_{input})$$

The weights *w_i_* are determined by the normalized amplitudes of the corresponding frequency components. Finally, the fused features are projected back to the same one-dimensional format as the input data through the dimension conversion module, completing the wind-power prediction task [29].

## 3. Complexity Analysis

The computational complexity of the DTCMMA model is mainly concentrated in the CMMA module, whose time complexity originates from the following three submodules. The computation of the channel attention module consists of three stages: feature aggregation, nonlinear transformation, and weight generation. The global mean pooling and global maximum pooling operations traverse the entire feature map with a complexity of O(H × W × D), while the two fully connected layers in the dual-path MLP network have complexities of O(D × D/r) and O(D/r × D), respectively. Therefore, the total complexity of the channel attention is O(H × W × D + D^2^/r). The spatial-attention module adopts a multiscale depthwise separable convolution design. The base 5 × 5 depthwise separable convolution has a complexity of O(25 × H × W × D). The three groups of cross-direction asymmetric convolutions have complexities of O(14 × H × W × D), O(22 × H × W × D), and O(42 × H × W × D), respectively. Adding the final 1 × 1 fusion convolution with a complexity of O(H × W × D), the total complexity of the spatial-attention module is O(104 × H × W × D). Although this complexity value is relatively high, depthwise separable convolutions have significant computational advantages over standard convolutions, and the asymmetric convolution kernel design avoids the computational waste caused by extensive padding operations in traditional Inception blocks. The pixel-attention module is implemented via a lightweight convolutional network. The first 1 × 1 convolution, which compresses channels, has a complexity of O(H × W × D^2^/8), and the second 1 × 1 convolution, mapping to a single channel, has a complexity of O(H × W × D/8). The total complexity is O(H × W × D^2^/8). Based on the above analysis, the overall time complexity of the SCSPA module is O(H × W × D × (105 + D/8) + D^2^/r), which significantly reduces computational complexity compared to the traditional full attention mechanism’s O((H × W)^2^ × D), especially when H × W is large. By introducing depthwise separable convolutions and channel compression techniques, the model achieves a substantial improvement in computational efficiency while maintaining strong feature representation capability.

## 4. Experimental Design

### 4.1. Data Selection

To ensure research reproducibility and result comparability, we provide comprehensive descriptions of all datasets used in our experiments. The dataset partitioning details are shown in Table 1.

Wind-Farm Datasets (WF-S1, WF-S2, WF-S3, WF-S4): Our wind-power datasets are derived from the Chinese State Grid Renewable Energy Generation Forecasting Competition dataset [30], which originally contained data from six wind farms across China, spanning 2019–2020. To facilitate direct comparison with ETT benchmark datasets and analyze performance differences across various data types, we extracted and reformatted four representative wind-farm subsets (WF-S1 through WF-S4) to match the temporal length of ETTh1 (17,421 data points). Each dataset maintains a 15 min temporal resolution and includes meteorological features such as wind speed at multiple heights, wind direction, ambient temperature, relative humidity, and atmospheric pressure, along with the corresponding power output measurements. The wind farms represent diverse geographical conditions across northern and northwestern China, including desert, mountainous, and plain terrains with nominal capacities ranging from 36 MW to 200 MW. Data preprocessing involved handling missing values (typically < 3% of total data) through linear interpolation and removing outliers using statistical filtering based on 3-sigma rules.

ETT Series Datasets: The Electricity Transformer Temperature (ETT) dataset [14] serves as a classic benchmark in time-series forecasting for energy systems. This dataset comprises four subsets: ETTh1 and ETTh2 (hourly measurements) and ETTm1 and ETTm2 (15 min measurements). The data originates from electricity transformers in different regions and captures transformer oil temperature as the target variable along with six electrical load-related features as covariates. The temporal coverage spans from July 2016 to July 2018, providing approximately two years of continuous measurements. ETTh1 and ETTh2 contain 17,420 hourly observations each, while ETTm1 and ETTm2 include 69,680 quarter-hourly records. These datasets are particularly valuable for evaluating model performance on power-system operational data, which shares similar characteristics with renewable energy integration challenges in modern grids.

Weather Dataset: The Weather dataset [31] contains comprehensive meteorological observations collected over multiple years, featuring 21 distinct weather parameters that directly influence renewable energy generation patterns. Variables include temperature, humidity, wind speed, wind direction, atmospheric pressure, solar radiation intensity, precipitation levels, and derived weather indices. The dataset employs 10 min sampling intervals, providing high-resolution temporal granularity suitable for short-term forecasting applications. Geographic coverage encompasses temperate climate zones with seasonal variations that create diverse weather patterns throughout the data collection period. This dataset serves as an important testbed for evaluating DTCMMA’s capability to handle complex multidimensional meteorological time series that exhibit strong seasonal and diurnal patterns characteristic of weather systems.

(1) The dataset [32] originates from the 2021 Renewable Energy Power Forecasting Competition organized by the State Grid Corporation of China. It covers six wind farms across China, including desert, mountainous, and plain terrains, spanning various climatic and geographical conditions. We selected four of the six datasets. The wind farms are equipped with extensive monitoring and data acquisition systems, featuring a large number of sensors and sampling devices. These systems are capable of recording and reflecting both the operational status of wind turbines and the surrounding environmental changes.
(2) Weather dataset: This dataset includes 21 meteorological features such as temperature, humidity, and wind speed, all of which directly impact the power output of wind turbines. It is used to validate the model’s effectiveness in processing multidimensional meteorological time-series data.
(3) ETT series dataset [22]: This dataset includes four subsets—ETTh1, ETTh2, ETTm1, and ETTm2. As a classical benchmark in the power systems domain, it records transformer temperature, electrical load, and other operational states of power equipment. Given its close relevance to power transmission and grid integration in wind farms, it is suitable for evaluating the generalization capability of the model in power-system time-series forecasting tasks.

### 4.2. Baseline Model

The TCMMA model is compared with eight baseline models, five of which are Transformer and its variants: Transformer [13], Reformer [32], Informer [14], Autoformer [15], and the latest iTransformer [16]; one of which pioneered the first time-series dataset in the TimesNet for dimensional transformation [4]; and one time-series forecasting architecture, TimeMixer [18], that employs a decomposition mixing strategy. For the baseline models, we use the proposed settings described in the respective papers.

Transformer: a pioneering sequence modeling framework with a pure attention mechanism, which discards the traditional recurrent neural network structure and employs a multi-head self-attention mechanism to construct the encoder-decoder system. It realizes parallelized training and global dependency capture.

Reformer: Aimed at the memory bottleneck problem of Transformer when dealing with ultra-long sequences. Through technical innovations such as locally sensitive hash attention and a reversible residual layer, memory consumption is greatly reduced while maintaining the expressive ability of the model, which enables the deep network to efficiently deal with long-distance sequences.

Informer: Innovatively introduces the probabilistic sparse self-attention mechanism, reducing the quadratic complexity of attention computation by dynamically selecting key Query-Key pairs. It also designs the distillation operation and generative decoding strategy, which effectively solves the problem of balancing computational efficiency with prediction accuracy in long sequence prediction.

Autoformer: The idea of sequence decomposition is deeply integrated into the Transformer architecture, and the embedded decomposition module realizes the progressive modeling of trend and seasonal components and proposes a new attention mechanism based on sequence autocorrelation, which is more suitable for capturing the cyclical dependence of the time series than the traditional dot product attention.

iTransformer: A disruptive inverted Transformer architecture is proposed, which converts traditional time-dimensional attention computation into variable-dimensional attention computation, thereby better capturing cross-variable interaction patterns and complex correlations in multivariate time series by establishing dependencies between variables instead of time steps.

TimesNet: Based on the inherent multi-periodicity of time series, we construct a time-series modeling paradigm with a two-dimensional perspective, capturing the complex time-varying patterns within and between periods in the transformed two-dimensional representation space via Inception blocks.

TimeMixer: A time-series forecasting architecture using decomposition and mixing strategy, which divides the forecasting task into two complementary phases—past information mixing and future information mixing—and effectively integrates historical information of different time scales through the multiscale patch mixing mechanism to realize more accurate long-term forecasts.

Implementation details: We use standard hyperparameter settings across all datasets: a batch size of 32, a learning rate of 1 × 10^−4^, and training for 10 epochs with a weight decay factor of 0.95 applied at each epoch [33,34]. The ADAM optimizer and cosine annealing scheduler [35,36,37] are employed. Each dataset is trained for 200 iterations. All experiments are implemented using PyTorch2.0.0, and MSE (mean square error) and MAE (mean absolute error) are adopted as evaluation metrics [38], where Ŷ denotes the predicted result, Y is the ground truth, and *n* is the number of samples.

### 4.3. Long Time-Series Forecasting

In the long time-series prediction task, we design the experiment to take an input sequence length of 96 to predict sequences with lengths of 96, 192, 336, and 720, respectively. We consider that, firstly, the input sequence length of 96 can provide the model with long enough historical information to capture the main periodicity and trend changes in the time series, which lays the foundation for accurate prediction. The prediction length, from 96 to 720, can fully test the model’s prediction ability for different time scales, including both relatively short medium-term forecasts and longer-term prediction scenarios, which is consistent with the demand for wind-power prediction in practical applications. For example, short-term wind-power prediction can be used for daily scheduling of the grid, while longer-term prediction can help develop a more macro energy planning and equipment maintenance program.

### 4.4. Ablation Experiment

In order to validate the effectiveness of each component in the DTCMMA model, especially the contribution of different attention sub-modules in the CMMA synergistic multidimensional and multiscale attention mechanism, detailed ablation experiments are designed in this section using the WDsite1 dataset. The impact of each component on the overall performance of the model is analyzed by gradually removing and adding different attention modules. The ablation experiments were designed for the following four model variants:

- DTCMMA-C: DTCMMA model with only the channel attention module retained;
- DTCMMA-S: DTCMMA model retaining only the spatial-attention module;
- DTCMMA-P: DTCMMA model retaining only the pixel-attention module;
- DTCMMA-Full: The full DTCMMA model (with three attention modules).

All variants keep the same dimension transformation module and underlying network architecture, with adjustments only in the CMMA attention mechanism part. The experiments use the same data division, training parameters, and evaluation metrics as the main experiment to ensure comparable results.

## 5. Results and Analysis

### 5.1. Analysis of the Results of the Long-Series Prediction Task

The experimental findings and their associated statistical analyses are delineated in Table 2, Table 3, Table 4 and Table 5. Table 2 presents the experimental results for the long-series prediction task. In this paper, mean square error (MSE) and mean absolute error (MAE) are chosen as performance evaluation criteria, and a comprehensive comparative performance analysis is carried out under multiple prediction time windows. The experimental results show that the DTCMMA model achieves significant performance advantages over the seven comparative baseline models on the four wind-farm datasets, ETT, and Weather datasets. Specific analyses show the following: (1) DTCMMA achieves a significant improvement in MSE and MAE metrics with an average improvement of 34.22% and 27.67%, respectively, compared to the traditional Transformer. The main limitations of the Transformer architecture are its O(L^2^) quadratic computational complexity, which is inefficient in dealing with long sequences, and its global attention-based design, which makes it difficult to effectively capture local temporal patterns and periodic features in time series. DTCMMA can better encode intra-periodic and inter-periodic variability patterns by explicitly reconstructing one-dimensional temporal sequences into two-dimensional representations through a dimension transformation strategy. (2) Compared to iTransformer, DTCMMA improves the performance by 2.57% and 2.67% on average. iTransformer converts the attention computation from the time dimension to the variable dimension by structural inversion, i.e., establishing the attention relationship between multivariate variables rather than between time steps. Although this design can better capture the interdependence between variables, it is still essentially a one-dimensional sequence modeling paradigm, which is not able to take full advantage of the multiscale periodic structural information in the time-series data. The inverted architecture still faces the challenges of computational efficiency and local temporal pattern capture when dealing with long sequences, whereas DTCMMA’s 2D reconstruction strategy, combined with the collaborative multidimensional attention mechanism, is able to simultaneously model complex dependencies in both temporal and variable dimensions. (3) DTCMMA achieves a performance improvement of 6.19% and 5.97% when compared with TimesNet, which also adopts 2D reconstruction. Although it also adopts a 2D reconstruction strategy, the standard Inception block it uses is dominated by square convolutional kernels [39], and this design requires a large number of padding operations when dealing with long stripes of data formed after period segmentation, which not only increases the computational burden, but also may introduce noisy information [40,41,42]. In contrast, the asymmetric convolutional kernels and cross-directional convolutions specifically designed for DTCMMA are able to better adapt to the geometric properties of the reconstructed data, thus achieving more effective feature extraction and spatiotemporal dependency modeling.

### 5.2. Analysis of Ablation Experiment Results

The ablation experiments verified the effectiveness and synergistic importance of the three attention mechanisms in the DTCMMA model. The experimental results show that the complete DTCMMA model (All) achieves the optimal MSE and MAE performance across all prediction lengths for both WF-S1 and WF-S4 datasets, which fully proves the superiority of synergistic multidimensional and multiscale attention mechanisms.

We can find significant differences in the degree of contribution of different attention mechanisms by comparatively analyzing the performance changes when each single attention module is missing. The absence of spatial attention (without SA) has the most significant negative impact on model performance, with an average increase of approximately 4.2% in MSE after removing spatial attention from the WF-S1 dataset. This suggests that the spatial-attention mechanism plays a key role in capturing inter-location dependencies in 2D reconstructed data. The removal of pixel attention (without PA) similarly led to a performance degradation, with an average MSE increase of about 1.8%, which demonstrates the importance of learning fine-grained features for the data. In contrast, the absence of channel attention (without CA) caused a relatively small performance degradation, but still resulted in some accuracy loss. The detailed ablation experimental results are shown in Table 6.

Of particular note, the advantage of the full model over the missing single-attention mechanism variant is more pronounced as the prediction length increases (from 96 to 720), suggesting that the synergy of the three attention mechanisms provides greater modeling power and stability in long-term prediction tasks and is better able to deal with the complex spatiotemporal dependencies in wind-power data.

We conducted a rigorous statistical analysis to evaluate DTCMMA’s performance. Specifically, for each dataset-horizon combination, we performed experiments using three independent random seeds, then averaged the results from these three trials to obtain 32 final samples (8 datasets × 4 horizons) for paired t-tests. Key findings include that DTCMMA significantly outperforms iTransformer, with a 3.60% MSE reduction (t = −4.41, *p* < 0.001, Cohen’s d = 0.78), demonstrating meaningful improvement over established Transformer variants. Compared to TimeMixer, DTCMMA shows equivalent performance with a minimal 0.22% difference (t = −0.63, *p* = 0.534), indicating our method achieves state-of-the-art performance levels.

**Table 3:** Results of the long time-series prediction task for the ETT and Weather datasets. (Red represents the best/optimal, blue represents the second best/sub-optimal).

Metric		DTCMMA		TimeMixer		iTransformer		TimesNet		Reformer		Autoformer		Informer		Transformer	
		MSE	MAE	MSE	MAE	MSE	MAE	MSE	MAE	MSE	MAE	MSE	MAE	MSE	MAE	MSE	MAE
ETTm1	96	0.332	0.356	0.320	0.357	0.344	0.378	0.338	0.375	0.538	0.528	0.505	0.475	0.672	0.571	0.593	0.529
	192	0.382	0.382	0.362	0.382	0.383	0.396	0.374	0.387	0.658	0.592	0.553	0.496	0.795	0.669	0.719	0.595
	336	0.412	0.402	0.396	0.406	0.418	0.418	0.410	0.411	0.898	0.721	0.621	0.537	1.212	0.871	0.860	0.680
	720	0.466	0.437	0.468	0.445	0.487	0.457	0.478	0.450	1.102	0.841	0.671	0.561	1.166	0.823	0.933	0.724
	Average	0.398	0.394	0.384	0.397	0.408	0.412	0.400	0.406	0.799	0.671	0.588	0.517	0.961	0.734	0.776	0.632
ETTm2	96	0.180	0.258	0.180	0.259	0.186	0.272	0.187	0.267	0.658	0.619	0.255	0.339	0.365	0.453	0.326	0.426
	192	0.246	0.299	0.247	0.303	0.254	0.314	0.249	0.309	1.078	0.827	0.450	0.340	0.533	0.563	0.559	0.576
	336	0.306	0.337	0.307	0.339	0.316	0.351	0.321	0.351	1.549	0.972	0.339	0.372	1.363	0.887	0.795	0.635
	720	0.413	0.399	0.396	0.399	0.414	0.407	0.408	0.403	2.631	1.242	0.433	0.432	3.379	1.388	1.855	1.080
	Average	0.286	0.324	0.279	0.325	0.293	0.336	0.291	0.333	1.479	0.915	0.327	0.371	1.410	0.823	0.884	0.679
ETTh1	96	0.381	0.393	0.384	0.400	0.391	0.406	0.384	0.402	0.837	0.728	0.449	0.459	0.865	0.713	0.396	0.404
	192	0.429	0.421	0.437	0.429	0.441	0.436	0.436	0.429	0.923	0.766	0.500	0.482	1.008	0.792	0.444	0.430
	336	0.473	0.442	0.473	0.437	0.491	0.462	0.491	0.469	1.097	0.835	0.505	0.484	1.128	0.873	0.493	0.458
	720	0.470	0.467	0.586	0.446	0.509	0.494	0.521	0.500	1.257	0.889	0.498	0.500	1.215	0.896	0.520	0.489
	Average	0.438	0.431	0.470	0.451	0.458	0.450	0.458	0.450	1.029	0.805	0.488	0.481	1.054	0.819	0.463	0.445
ETTh2	96	0.304	0.347	0.297	0.348	0.301	0.350	0.340	0.374	2.626	1.317	0.346	0.388	3.755	1.525	1.141	0.802
	192	0.389	0.398	0.369	0.392	0.381	0.400	0.402	0.414	11.120	2.979	0.456	0.452	5.602	1.931	2.032	1.051
	336	0.427	0.431	0.427	0.432	0.424	0.432	0.452	0.452	9.323	2.769	0.471	0.475	2.723	1.340	2.474	1.197
	720	0.403	0.427	0.427	0.445	0.430	0.447	0.462	0.468	3.874	1.697	0.474	0.484	3.467	1.473	2.086	1.151
	Average	0.381	0.401	0.380	0.404	0.384	0.407	0.414	0.427	6.735	2.190	0.437	0.450	3.887	1.567	1.993	1.050
Weather	96	0.162	0.209	0.163	0.209	0.176	0.216	0.172	0.220	0.689	0.596	0.266	0.336	0.300	0.384	0.404	0.460
	192	0.217	0.251	0.209	0.252	0.225	0.258	0.219	0.261	0.752	0.638	0.307	0.367	0.598	0.544	0.601	0.596
	336	0.277	0.297	0.264	0.293	0.281	0.300	0.280	0.306	0.639	0.596	0.359	0.395	0.702	0.620	0.359	0.418
	720	0.342	0.341	0.345	0.345	0.359	0.350	0.365	0.359	1.130	0.792	0.419	0.428	0.831	0.731	0.416	0.449
	Average	0.249	0.275	0.245	0.275	0.260	0.281	0.259	0.286	0.803	0.656	0.338	0.382	0.608	0.570	0.445	0.481

DTCMMA exhibits the smallest variance (0.200) among all methods, confirming stable performance across diverse experimental conditions. These results provide strong statistical evidence for the effectiveness of our collaborative multidimensional attention mechanism while maintaining competitive performance with recent advances in time-series forecasting.

**Table 4:** Results of the statistical analysis of experimental findings. (Red represents the best/optimal, blue represents the second best/sub-optimal).

Metric	DTCMMA		TimeMixer		iTransformer		TimesNet		Reformer		Autoformer		Informer		Transformer	
	MSE	MAE	MSE	MAE	MSE	MAE	MSE	MAE	MSE	MAE	MSE	MAE	MSE	MAE	MSE	MAE
ETTm1	0.398	0.394	0.384	0.397	0.408	0.412	0.400	0.406	0.799	0.671	0.588	0.517	0.961	0.734	0.776	0.632
ETTm2	0.286	0.324	0.279	0.325	0.293	0.336	0.291	0.333	1.479	0.915	0.327	0.371	1.410	0.823	0.884	0.679
ETTh1	0.438	0.431	0.470	0.451	0.458	0.450	0.458	0.450	1.029	0.805	0.488	0.481	1.054	0.819	0.463	0.445
ETTh1	0.381	0.401	0.378	0.391	0.384	0.407	0.414	0.427	6.735	2.190	0.437	0.450	3.887	1.567	1.993	1.050
WF-S1	1.571	0.882	1.587	0.888	1.613	0.903	1.651	0.908	2.177	1.133	1.680	0.953	1.610	0.941	1.636	0.946
WF-S2	0.747	0.640	0.733	0.643	0.764	0.655	0.881	0.694	1.078	0.833	0.815	0.700	0.846	0.736	0.808	0.709
WF-S3	1.155	0.790	1.128	0.788	1.178	0.807	1.155	0.836	1.592	0.971	1.246	0.858	1.606	0.984	1.419	0.928
WF-S4	1.147	0.788	1.149	0.796	1.182	0.808	1.283	0.896	1.652	0.992	1.289	0.878	1.572	0.978	1.310	0.937
Weather	0.249	0.275	0.245	0.275	0.260	0.281	0.259	0.286	0.803	0.656	0.338	0.382	0.608	0.570	0.445	0.481
Average	0.455	0.290	0.456	0.293	0.472	0.298	0.507	0.325	2.245	0.514	0.520	0.340	0.985	0.425	0.574	0.377
Variance	0.200	0.047	0.201	0.047	0.211	0.048	0.228	0.056	3.070	0.192	0.214	0.046	0.827	0.071	0.256	0.042

**Table 5:** Results of the significance test.

Comparison Group	Mean MSE	Std. Dev	t-Value	Two-Tailed *p*-Value	Mean Relative Reduction
DTCMMA vs. iTransformer	−0.017	0.0218	−4.41	<0.001	3.60%
DTCMMA vs. TimeMixer	−0.001	0.0089	−0.63	0.534	0.22%

**Table 6:** Results of ablation experiments. (Red represents the best/optimal).

Model		All		Without CA		Without SA		Without PA	
Metric		MSE	MAE	MSE	MAE	MSE	MAE	MSE	MAE
**WF-S1**	96	1.178	0.738	1.177	0.737	1.164	0.735	1.181	0.738
**WF-S1**	192	1.571	0.882	1.571	0.882	1.648	0.901	1.607	0.893
**WF-S1**	336	1.734	0.947	1.722	0.948	1.785	0.958	1.757	0.951
**WF-S1**	720	1.800	0.963	1.801	0.957	1.790	0.962	1.827	0.969
**WF-S4**	96	0.871	0.670	0.874	0.671	0.889	0.671	0.881	0.673
**WF-S4**	192	1.178	0.789	1.178	0.789	1.194	0.793	1.182	0.791
**WF-S4**	336	1.277	0.840	1.278	0.840	1.276	0.838	1.279	0.841
**WF-S4**	720	1.261	0.852	1.262	0.854	1.265	0.854	1.275	0.859

### 5.3. Analysis of Model Performance in Unusual Seasonal Patterns

While DTCMMA demonstrates robust performance across standard seasonal patterns, it is important to acknowledge its potential limitations when dealing with unusual or irregular seasonal cycles that deviate from typical periodic structures. The core limitation stems from the inherent constraints of fast Fourier transform (FFT) in the dimensional transformation module.

Fundamental FFT Limitations and Time-Frequency Localization Issues: The most critical weakness of our approach lies in FFT’s inability to provide temporal information about frequency components. While FFT excels at identifying the dominant frequencies present in wind-power data, it lacks the fundamental capability to determine when these frequencies occur within the time series. This limitation becomes particularly problematic when dealing with non-stationary seasonal patterns, where the dominant periods may shift over time. For instance, during transitional seasons (spring and autumn), wind farms often experience evolving wind patterns where the dominant 24 h diurnal cycle may gradually shift to include stronger weekly or monthly components. Since FFT provides only frequency domain information without temporal localization, DTCMMA cannot adapt to these time-varying periodic structures dynamically. The model essentially assumes that the identified periodic components remain constant throughout the entire sequence, which is a significant oversimplification for real-world wind-power scenarios.

Challenges with Non-Standard Periodicity: This FFT limitation manifests in several critical scenarios. First, irregular seasonal patterns caused by climate anomalies (such as the El Niño or La Niña phenomena) introduce time-varying periodicity that FFT cannot capture. The algorithm identifies average frequency components across the entire time series but cannot detect when these patterns begin, intensify, or fade away. Second, when multiple overlapping periods with similar frequencies exist, the Top-K selection mechanism, combined with FFT’s temporal blindness, may result in selecting frequencies that are dominant only in certain time segments while missing critical periods that occur sporadically. Third, for wind farms experiencing gradual environmental changes or seasonal transitions, the underlying periodic patterns evolve continuously, but FFT can only provide a static snapshot of the frequency content.

## 6. Visualization and Performance Analysis

**Figure 4:**
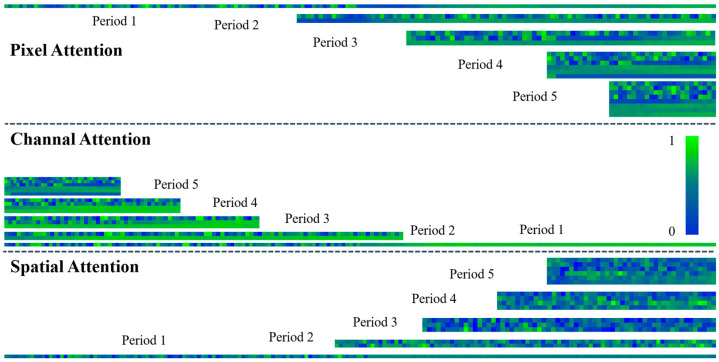
Pixel, channel, and spatial convolutional attention module output feature visualization map.

As a core component of the DTCMMA model, the synergistic multidimensional multiscale attention mechanism plays a key role in capturing the complex spatiotemporal dependencies in wind-power time-series data. In order to deeply analyze the feature learning capability of the proposed channel–space–pixel multidimensional attention mechanism based on a convolutional neural network, we systematically visualize the weight distributions generated by the three attention modules, and the experimental results are shown in Figure 4. The visualization results clearly show the differential importance weight distributions learned by the model at different feature channels, spatial locations, and pixel levels, which verifies that the proposed multidimensional attention mechanism is able to effectively identify and model the multilevel complex dependencies in wind-power data. The visualization results are shown in Figure 4.

**Figure 5:**
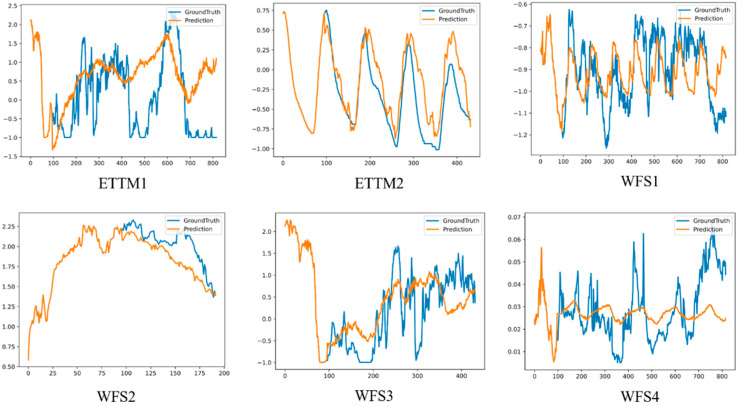
Fitting effect of DTCMMA model’s predicted values vs. true values.

In order to more intuitively demonstrate the excellent prediction effect of our model, we also designed a visualization experiment comparing the predicted value with the real value, and the fitting results are shown in Figure 5. As shown in the visualization results of Figure 5, the DTCMMA model demonstrates excellent prediction performance. The model can accurately learn the trend variations in the data, particularly excelling on several wind-power datasets where the predicted curves closely align with the ground truth values. Notably, on the ETTm2 dataset, the model not only accurately captures the overall trends but also precisely predicts each fluctuation pattern, demonstrating good fine-grained forecasting capability. However, there are still some deficiencies in predicting detailed variations on the ETTm1 and WF-4 datasets, especially when dealing with local sudden changes and subtle fluctuations, where certain deviations exist between predicted and actual values, providing directions for future model optimization.

**Figure 6:**
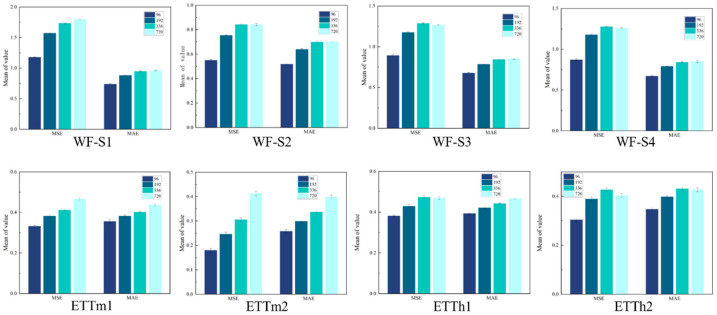
MSE and MAE visualization of the DTCMMA model on eight datasets.

To verify the robustness of the model, we conducted experiments using three different random seeds as noise [43]. We obtained the errors for six datasets at different prediction lengths {96, 192, 336, and 720} to assess the stability and reliability of the model in the face of random noise, and the results are shown in Figure 6.

Due to our innovative attention-mechanism design for the wind-power dataset, the DTCMMA model has a significant advantage over other baseline models in terms of time efficiency, which is three times that of iTransformer, the latest improved model of the Transformer family of models, and eight times that of the TimesNet model.

**Figure 7:**
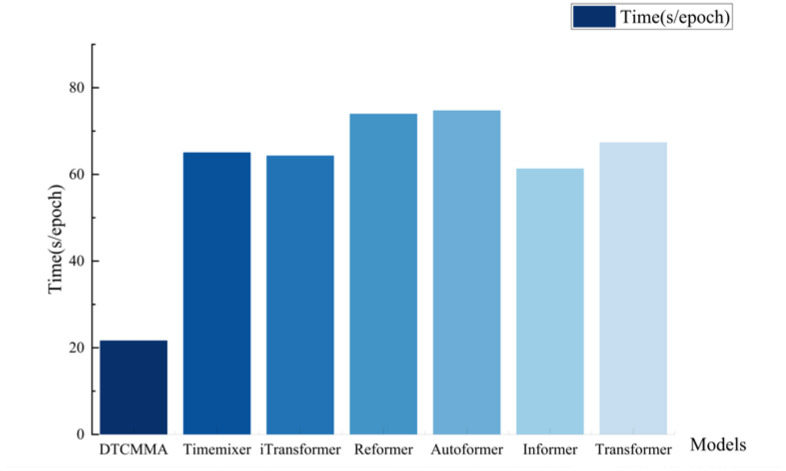
Schematic representation of training speed for eight models.

**Figure 8:**
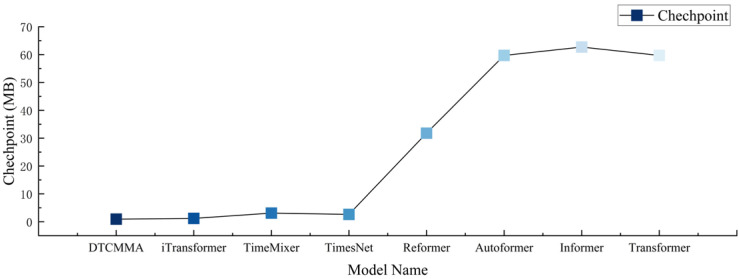
Schematic representation of the size of the number of weighted parameters for eight models.

The speed of our model versus the baseline model is plotted against the number of parameters, as shown in Figure 7 and Figure 8. The DTCMMA model has only a small number of parameters, which results in a more than three-fold increase in training speed compared to the baseline model.

**Figure 9:**
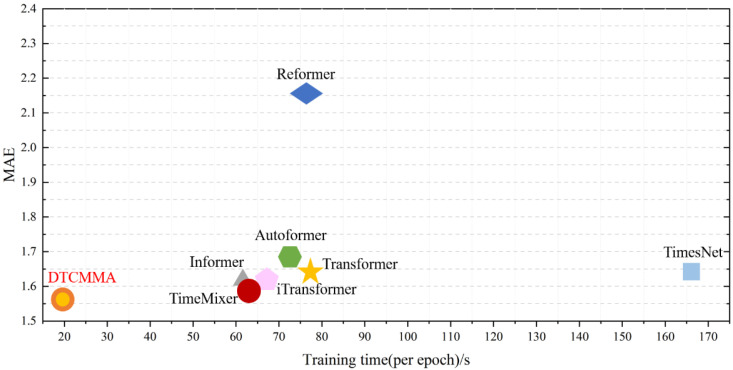
Comparison of prediction accuracy and speed for eight models.

In our experimental design, to facilitate understanding of training quality and model performance, we incorporate the calculation and display of multiple evaluation metrics, including MSE, MAE, RMSE, and RMAE, after each epoch of training. Therefore, the recorded total duration includes not only the actual model training time but also the computation time for these evaluation metrics, where the latter does not truly reflect the model’s training efficiency. Since we typically set each experiment to train for 10 epochs, using “s/epoch” as the measurement method can more accurately reflect the actual time consumption of a single training cycle, excluding the interference of metric calculations and other additional operations on time measurement. This standardized time measurement approach enables a fairer comparison of training speeds across different models. As shown in the visualization results of Figure 9.

We chose to conduct speed visualization experiments on the WF-S3 dataset with an input length of 96 and a prediction output length of 96. The experimental results show that our DTCMMA model achieves a training speed of 21.58 s/epoch, while the second-best TimeMixer model requires 64.98 s/epoch, making our model three times faster. This fully demonstrates that our proposed collaborative multidimensional multiscale attention mechanism achieves significant computational efficiency advantages while maintaining high prediction accuracy.

## 7. Limitations and Challenges of the DTCMMA Model

While DTCMMA demonstrates superior performance across multiple datasets and prediction tasks, it is essential to acknowledge the inherent limitations and challenges associated with our proposed approach, particularly those stemming from the fundamental constraints of the fast Fourier transform-based dimensional transformation strategy.

FFT’s Temporal Blindness and Its Cascading Effects: The most significant architectural limitation of DTCMMA lies in its reliance on fast Fourier transform for period identification, which suffers from the fundamental inability to provide temporal localization of frequency components [44]. While FFT effectively identifies which frequencies are present in the wind-power time series, it cannot determine when these frequencies occur, leading to several critical issues [45]. This temporal blindness means that our dimensional transformation assumes static periodicity throughout the entire input sequence, which is unrealistic for real-world wind-power data that exhibits time-varying seasonal patterns, equipment-dependent variations, and weather-induced period shifts. The cascading effect of this limitation is that the 2D reconstruction may be based on periodic assumptions that are only valid for certain portions of the input sequence, potentially leading to suboptimal feature representations and reduced prediction accuracy during periods of transitional or evolving seasonality.

## 8. Future Research Directions and Development Plans

Cross-Domain Dataset Validation: Future work will focus on evaluating DTCMMA’s performance across diverse time-series forecasting domains beyond wind-power applications. We plan to conduct comprehensive experiments on financial time series (e.g., stock prices, cryptocurrency data), medical signal analysis (e.g., ECG, EEG recordings), traffic flow prediction, and industrial sensor data. These experiments will help identify the model’s generalization capabilities and domain-specific limitations, potentially revealing the need for adaptive architectural modifications or domain-specific preprocessing techniques to handle different data characteristics and temporal patterns.

Enhanced Dimensional Transformation Methods: To address the identified limitations of FFT-based period identification, we will explore alternative time-frequency analysis techniques that can provide both frequency and temporal localization information [46]. Planned approaches include implementing continuous wavelet transforms (CWT), empirical mode decomposition (EMD), and variational mode decomposition (VMD) for more robust period detection. These methods may better capture time-varying periodicities and irregular seasonal patterns, potentially improving the model’s adaptability to non-stationary time-series data with evolving temporal structures.

Advanced Feature Extraction Architectures: We will investigate more sophisticated feature extraction modules to better capture complex temporal dependencies in time-series data. This includes exploring transformer-based alternatives to the current convolutional attention mechanisms, implementing graph neural networks to model inter-variable relationships, and developing adaptive attention mechanisms that can dynamically adjust their focus based on the temporal characteristics of the input data. Additionally, we plan to experiment with self-supervised learning approaches to pre-train feature extractors on large-scale unlabeled time-series data, potentially improving the model’s ability to learn meaningful temporal representations.

## 9. Conclusions

In this paper, an efficient wind-power prediction method based on the combination of dimensional transformation and synergistic multidimensional multiscale attention mechanism, called DTCMMA, is proposed. The method automatically identifies the major periodic components of a time series using the dimensional transformation module, which employs fast Fourier transform, and reconstructs the one-dimensional time series as a two-dimensional representation, which efficiently encodes the intra-periodic and inter-periodic variability patterns.

The CMMA synergistic multidimensional and multiscale attention mechanism significantly enhances the model’s ability to capture complex spatiotemporal dependencies through the hierarchical fusion of channel attention, spatial attention, and pixel attention. Aiming at the long-strip geometry characteristic of 2D reconstructed data, the innovative design of asymmetric convolution kernel and cross-direction convolution effectively avoids the filling problem of the traditional square convolution kernel. The experimental results show that, compared with mainstream time-series prediction models, DTCMMA achieves significant performance improvement on multiple wind-farm datasets, realizing 24.6%, 15.2%, and 6.3% MSE improvement compared with Transformer, iTransformer, and TimesNet, respectively. Especially in the long-term prediction task, the proposed synergistic multidimensional attention mechanism demonstrates excellent adaptive ability and stable performance across datasets, providing a new technical path in the field of wind-power prediction.

Future research will focus on further optimizing the cycle discovery algorithm for more complex multi-period nested structures. Although the model proves good prediction stability on most datasets, there is still room for improvement in certain extreme weather scenarios and ultra-long-term prediction tasks. Therefore, it is necessary to further design more robust multiscale fusion mechanisms and adaptive weight adjustment strategies to adapt to the data characteristics of different wind farms and the dynamically changing needs of the actual industrial environment, thereby providing more reliable technical support for the stable operation of new energy power systems.

## Figures and Tables

**Table 1:** Dataset Introduction Table.

Dataset	Train	Vaild	Test	Dim	Split	Frequency
WF-S1	8545	2881	2881	11	6:02:02	15 min
WF-S2	8545	2881	2881	11	6:02:02	15 min
WF-S3	8545	2881	2881	12	6:02:02	15 min
WF-S4	8545	2881	2881	12	6:02:02	15 min
Weather	36,792	5271	10,540	21	7:01:02	10 min
ETTh1, ETTm2	8545	2881	2881	7	6:02:02	Hour
ETTm1, ETTm2	34,465	11,521	11,521	7	6:02:02	15 min

**Table 2:** Results of the power prediction task for a long time series with the wind dataset. (Red represents the best/optimal, blue represents the second best/sub-optimal.)

Metric		DTCMMA		TimeMixer		iTransformer		TimesNet		Reformer		Autoformer		Informer		Transformer	
		MSE	MAE	MSE	MAE	MSE	MAE	MSE	MAE	MSE	MAE	MSE	MAE	MSE	MAE	MSE	MAE
WF-S1	96	1.178	0.738	1.180	0.739	1.179	0.748	1.291	0.770	1.803	1.024	1.413	0.861	1.223	0.820	1.212	0.814
	192	1.571	0.882	1.552	0.880	1.619	0.907	1.634	0.901	2.106	1.104	1.684	0.958	1.578	0.953	1.334	0.833
	336	1.734	0.947	1.792	0.965	1.794	0.973	1.815	0.983	2.131	1.126	1.823	0.999	1.764	0.956	1.968	1.066
	720	1.800	0.963	1.824	0.969	1.859	0.983	1.862	0.977	2.667	1.279	1.798	0.995	1.875	1.033	2.032	1.071
	Average	1.571	0.882	1.587	0.888	1.613	0.903	1.651	0.908	2.177	1.133	1.680	0.953	1.610	0.941	1.636	0.946
WF-S2	96	0.550	0.519	0.540	0.527	0.590	0.555	0.624	0.559	0.866	0.767	0.721	0.646	0.635	0.615	0.551	0.571
	192	0.754	0.638	0.721	0.634	0.757	0.641	0.952	0.719	0.878	0.731	0.788	0.683	0.841	0.754	0.585	0.594
	336	0.842	0.700	0.826	0.700	0.843	0.705	0.950	0.737	1.086	0.841	0.874	0.733	0.805	0.714	0.843	0.753
	720	0.842	0.702	0.845	0.710	0.866	0.721	0.998	0.760	1.480	0.993	0.875	0.736	1.101	0.862	1.255	0.917
	Average	0.747	0.640	0.733	0.643	0.764	0.655	0.881	0.694	1.078	0.833	0.815	0.700	0.846	0.736	0.808	0.709
WF-S3	96	0.892	0.677	0.854	0.663	0.886	0.677	0.971	0.712	1.195	0.835	1.106	0.796	1.681	1.001	1.275	0.869
	192	1.175	0.786	1.162	0.792	1.183	0.801	1.064	0.882	1.338	0.893	1.233	0.845	1.485	0.937	1.235	0.854
	336	1.287	0.843	1.251	0.837	1.353	0.873	1.298	0.896	1.897	1.076	1.376	0.913	1.577	0.995	1.625	0.992
	720	1.266	0.853	1.245	0.860	1.290	0.876	1.287	0.854	1.937	1.081	1.268	0.878	1.681	1.001	1.543	0.996
	Average	1.155	0.790	1.128	0.788	1.178	0.807	1.155	0.836	1.592	0.971	1.246	0.858	1.606	0.984	1.419	0.928
WF-S4	96	0.871	0.670	0.872	0.672	0.897	0.684	0.990	0.730	1.208	0.822	1.277	0.873	1.240	0.868	1.275	0.889
	192	1.178	0.789	1.172	0.797	1.217	0.810	1.267	0.841	1.420	0.932	1.238	0.846	1.330	0.910	1.284	0.885
	336	1.277	0.840	1.288	0.850	1.320	0.863	1.302	0.981	2.029	1.115	1.376	0.913	1.902	1.095	1.625	1.007
	720	1.261	0.852	1.264	0.864	1.295	0.875	1.574	1.032	1.952	1.100	1.264	0.878	1.815	1.041	1.054	0.966
	Average	1.147	0.788	1.149	0.796	1.182	0.808	1.283	0.896	1.652	0.992	1.289	0.878	1.572	0.978	1.310	0.937

## Data Availability

Data is contained within the article. The data presented in this study is available from the corresponding author upon reasonable request.
